# Risk of lung cancer in Parkinson's disease

**DOI:** 10.18632/oncotarget.12964

**Published:** 2016-10-27

**Authors:** Xin Xie, Xiaoguang Luo, Mingliang Xie, Yang Liu, Ting Wu

**Affiliations:** ^1^ Department of Neurology, The First Affiliated Hospital of China Medical University, Shenyang City 110001, PR China; ^2^ Clinics of the People's Armed Police Command College, Tianjin City, 300000, PR China

**Keywords:** lung neoplasms, meta-analysis, Parkinson's disease, risk

## Abstract

Recently, growing evidence has revealed the significant association between Parkinson's disease (PD) and cancer. However, controversy still exists concerning the association between PD and lung cancer. A comprehensive article search for relevant studies published was performed using the following online databases: PubMed, Web of Science and Embase up to August 31, 2016. The pooled risk ratio (RR) and their 95 % confidence intervals (CI) were calculated using the method of inverse variance with the random-effects model. Fifteen studies comprising 348,780 PD patients were included in this study. The pooled result indicated that patients with PD were significantly associated with a decreased risk of lung cancer (RR: 0.53, 95% CI: 0.41−0.70, *P* < 0.001). In addition, subgroup analyses performed in Western population also confirmed the significant inverse relationship between PD and risk of lung cancer (RR: 0.48, 95% CI: 0.39−0.60, *P* < 0.001). In the subgroup analysis, a reduced risk of lung cancer in PD patients from Western population was consistent regardless of study design, gender, or study quality. In conclusion, PD patients were significantly associated with a reduced risk of lung cancer in Western population. The relationship between them in Asian population needs to be confirmed by future studies.

## INTRODUCTION

Parkinson's disease (PD) is the second most common neurodegenerative disorders characterized by the selective dopaminergic cell loss in the substantia nigra, and the prevalence of PD of the entire population is about 0.3% in industrialized countries [1]. Most PD cases are sporadic and until now the exact etiology of PD still remains unknown.

It is all to know that cancer is a disease characterized by cell infinite proliferation and lack of apoptosis. Recently, growing evidence has revealed the significant association between PD and cancer [2, 3], and PD plays different roles in risk of different cancers. For instance, some studies reported that PD was significantly associated with an increased risk of melanoma [4], but with a decreased risk of bladder cancer [5]. However, controversy still exists concerning the association between PD and lung cancer [3, 6, 7]. On the other hand, there is some potential evidence to link PD with lung cancer. For instance, PD and lung cancer are both regarded as disease characterized by multi-factor disorders involving genomic and environmental factors. Mutation of the PD-associated gene PARK2 also can be observed in lung cancer [8]. Moreover, recent studies reported that smoking played different roles in risk of PD and lung cancer [9, 10]. Thus, this controversial relationship between PD and lung cancer catches our attention.

In this study, we included the cohort and case-control studies to perform a meta-analysis which aimed to explore the association between PD and risk of lung cancer.

## RESULTS

### Baseline characteristics of included studies

**Figure 1:**
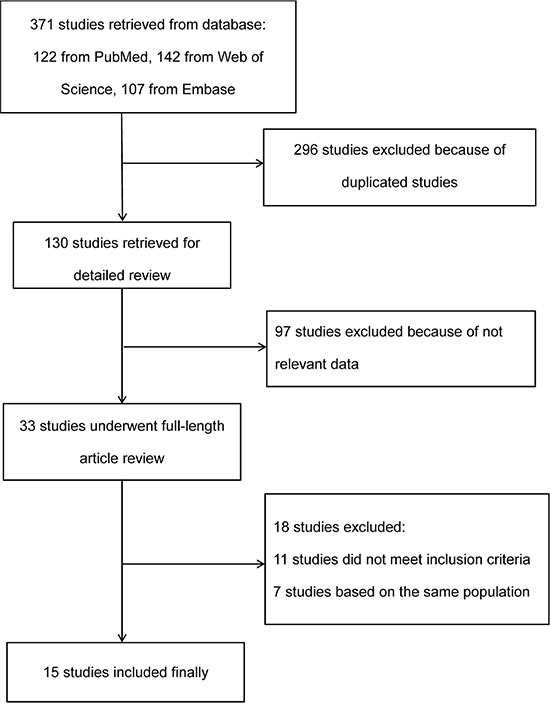
Flow diagram of study selection procedure

A total of 371 potentially relevant studies were identified in the database search. After exclusion of 296 duplicated studies, there were remain 130 studies. After title and abstract review, 97 studies were excluded. After full article review for the remaining 33 studies, 7 duplicated studies which were performed in the same population and 11 studies which did not meet inclusion criteria were excluded (Supplementary material 1). Finally, 15 studies were included in our meta-analysis [5–7, 11–22] (Figure 1).

These studies were published between 2000 and 2016, and contained 348,780 patients with PD. Among these 15 studies, 2 studies were performed in Asian population (1 in Japan and 1 in Taiwan), while 13 studies were conducted in Western population (6 in USA, 3 in UK, 1 in Canada, 1 in Denmark, 1 in Israel and 1 in Sweden). The baseline characteristics of each study are shown in Table 1.

**Table 1:** Baseline characteristics of included studies in this meta-analysis

Author	Year	Country /Area	Study design	Number of case (M/F), source	Number of control (M/F), source	Age of Case	Age of control	Adjustment	OI	SQ^a^
Minami [11]	2000	Japan	Cohort	228 (NR), medical institutions in Miyagi	NR, local population	NR	NR	NR	SIR	5
Elbaz [12]	2002	USA	Case-control	196 (121/75), Rochester Epidemiology Project	196 (NR), local population	71 (41–97)^b^	NR	age, sex	OR	7
Guttman [13]	2004	Canada	Cohort	15304 (NR), Ontario Health Insurance Plan	30608 (NR), general population	NR	NR	age, sex	RR	6
Driver [6]	2007	USA	Cohort	572 (NR), Physician's Health Study	478 (NR), Physician's Health Study	59.7 (39.8–85.0)^b^	59.8 (39.8-84.6)^b^	age	RR	6
Becker [14]	2010	UK	Cohort	2993 (NR), UK-based General Practice Research Database	3003 (NR), UK-based General Practice Research Database	NR	NR	age, sex, general practice, diagnosis date, years of history	IRR	6
Fois [15]	2010	UK	Cohort/Case-control	4355 (2150/2205),UK National Health Service hospitals	NR, UK National Health Service hospitals	NR	NR	age, sex, calendar year of first recorded admission	RR	7
Lo [16]	2010	USA	Cohort/Case-control	692 (433/259), Kaiser Permanente Northern California Medical Care Plan	761 (476/285), Kaiser Permanente Northern California Medical Care Plan	66.0±12.1	65.8±12.1	age, sex, cigarette smoking, alcohol consumption, body mass index	RR	7
Kareus [17]	2012	USA	Cohort	2998 (1886/1112), Utah Population Database	NR, Utah Population Database	NR	NR	age, sex, birth place	RR	5
Rugbjerg [18]	2012	Denmark	Cohort	20343(10712/9631), Danish Hospital Register	NR, general population	72.7^c^	NR	age, sex, calendar period	SIR	7
Wirdefeldt [19]	2013	Sweden	Cohort/Case-control	11786 (7135/4651), Swedish Patient Register	58930 (NR), Swedish Patient Register	62.5 ± 9.2	NR	sex, birth year	HR	7
Ong [5]	2014	UK	Cohort	219194 (NR), English national Hospital Episode Statistics	9015614 (NR), English national Hospital Episode Statistics	NR	NR	age, sex, calendar year, region of residence, quintile of patients	RR	8
Lin [7]	2015	Taiwan	Cohort	62023 (31486/30537), National Health Insurance	124046 (62972/61074), National Health Insurance	NR	NR	age, sex	HR	7
Freedman [20]	2016	USA	Case-control	NR, SEER-Medicare	NR, SEER-Medicare	NR	NR	age, sex, selection year	OR	5
Peretz [21]	2016	Israel	Cohort	7125 (3299/3826), Maccabi Health Services	NR, Maccabi Health Services	71.1 ± 10.6	NR	age, sex, chronological year	SIR	6
Tacik [22]	2016	USA	Case-control	971 (621/350), Mayo Clinic	478 (215/263), Mayo Clinic	67 (9–95)^b^	NR	age, sex	OR	5

Abbreviations, F:Female; HR: hazard ratio; IRR: incidence rate ratio; M:Male; NR: not report; OR: odds ratio; OI: outcome of interest; RR: relative risk; SEER: Surveillance, Epidemiology, and End Results; SIR: standardized incidence ratio; SQ: score of study quality.

a Study quality was judged based on the Newcastle-Ottawa Scale

b Median (Range)

c Mean age.

### PD and risk of lung cancer

**Figure 2:**
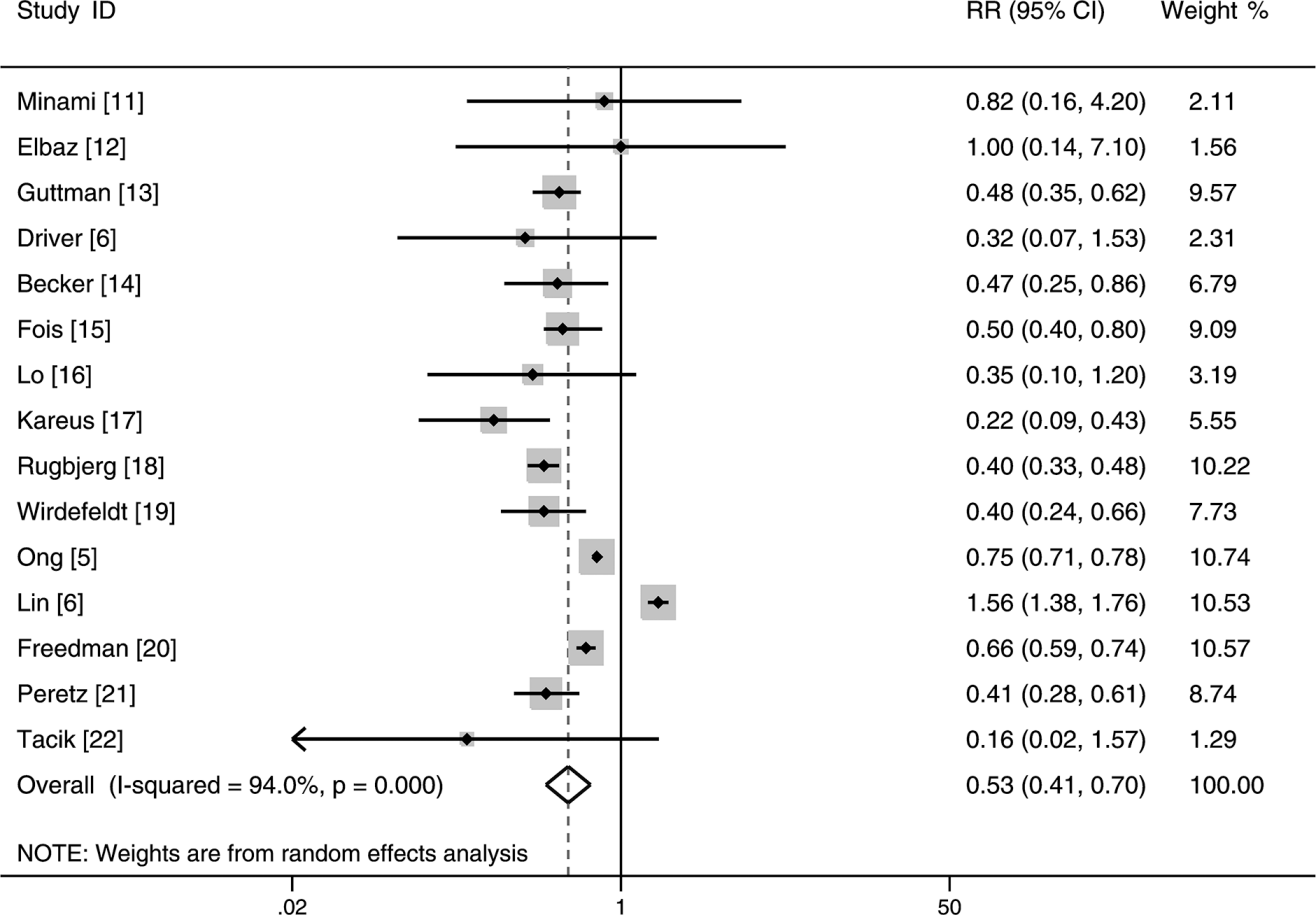
Forest plot of risk ratio for the association between Parkinson's disease and risk of lung cancer

The result of 12 cohort studies and 3 case-control studies indicated that the pooled RR of lung cancer in PD patients versus control patients was 0.53 (95% CI: 0.41–0.70, *P* < 0.001, *I*^2^ = 94%, Figure 2), thus showing that PD patients had a reduced risk of lung cancer.

In addition, we excluded two studies which were based on Asian population [7, 11] and performed further analyses in Western population. The pooled result showed that patients with PD were significantly associated with a decreased risk of lung cancer in Western population (RR: 0.48, 95% CI: 0.39–0.60, *P* < 0.001, *I*^2^ = 85%, Table 2). Furthermore, subgroup analyses revealed the significance of the inverse association between PD and risk of lung cancer was not affected by many factors such as study design, gender and study quality (Table 2).

**Table 2:** Subgroup analyses for association between Parkinson's disease and the risk of lung cancer in western population

Categories	*N*	Pooled RR	95% CI	*P* value	Heterogeneity	
					*I*^2^ (%)	Ph
Overall effect	13	0.48	0.39–0.60	< 0.001	84.9	< 0.001
Study design						
Cohort	10	0.45	0.33–0.60	< 0.001	88.3	< 0.001
Case-control	3	0.66	0.59–0.74	< 0.001	0	0.408
Gender						
Male	5	0.50	0.33–0.77	< 0.001	91.0	< 0.001
Female	5	0.56	0.41–0.78	< 0.001	68.0	0.010
Study quality						
> 6	6	0.50	0.34–0.75	0.001	90.3	< 0.001
≤ 6	7	0.46	0.34–0.61	< 0.001	66.1	0.007
The diagnosis time of LC						
Before PD	5	0.49	0.21–1.14	0.100	89.0	< 0.001
After PD	11	0.49	0.40–0.60	< 0.001	86.0	< 0.001

Abbreviations, CI: confidence interval; LC: lung cancer; N: number of studies; RR: risk ratio; PD: Parkinson's disease.

Ph: p value of Q test for heterogeneity test.

On the other hand, according to the diagnosis time of lung cancer, we divide the studies into before PD and after PD these two groups to perform subgroup analysis. The result indicated that the significant inverse relationship between PD and the risk of lung cancer could be found in after PD group (RR: 0.49, 95% CI: 0.40–0.60, *P* < 0.001), but it could not be found in before PD group (RR: 0.49, 95% CI: 0.21–1.14, *P* = 0.10, Table 2).

### Sensitivity analysis

In this study, sensitivity analysis was used to assess the stability of the results. The significant inverse association between PD and risk of lung cancer did not change in the sensitivity analysis, which removed each study in turn (Figure S1).

### Publication bias analysis

**Figure 3:**
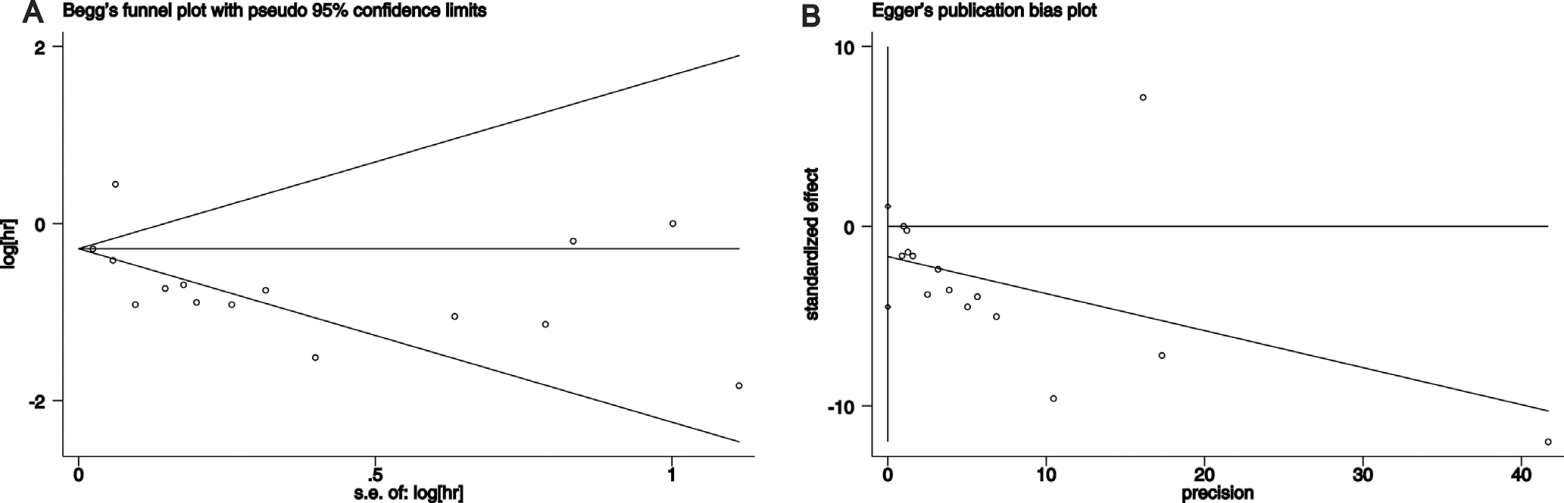
Test result for publication bias (**A**) Begg's test (*P* = 0.151); (**B**) Egger's test (*P* = 0.214).

In our meta-analysis, we used Begg's and Egger's tests to evaluate the effect of publication bias. The result indicated that there was no evidence of publication bias (Begg, *P* = 0.151; Egger, *P* = 0.214, Figure 3)

### Cumulative meta-analysis

We used cumulative meta-analysis to evaluate the association between PD and lung cancer risk with relation to publication year. Our result indicated that from 2004 to present, the significant inverse relationship between PD and risk of lung cancer remained consistent (Figure S2).

## DISCUSSION

To the best of our knowledge, this meta-analysis is the first study to systematically explore the association between PD and risk of lung cancer. In this study, we included 15 studies comprising 348,780 PD patients. The pooled result indicated PD patients had a 47% decrease in risk of developing lung cancer. Furthermore, we excluded two studies which were based on Asian population and conducted further subgroup analyses in Western population. The result also showed that patients with PD were significantly associated with a decreased risk of lung cancer in Western population. The significance of the inverse association between PD and risk of lung cancer was not affected by many factors such as study design, gender and study quality. The results of sensitivity analysis and cumulative meta-analysis, and the absence of publication bias also confirmed the robustness of the relationship between them.

There were several possible explanations for the correlation of PD and the reduced risk of lung cancer. First, different essential characteristics of these two diseases may be the primary cause. PD is a neurodegenerative disorders characterized by dopaminergic neuronal death, whereas lung cancer is a disease characterized by cell unlimited growth and lack of apoptosis. Cells in PD patients may be more likely to undergo apoptosis to fight against progression of cancer [5]. Second, the treatment of PD may provide another explanation. Dopamine, which is a drug used in the therapy for PD, was reported to be an effective anti-angiogenic drug for the treatment of malignant tumors [23], thus it may decease the risk of lung cancer. Indeed, our result indicated that the significant inverse relationship between PD and risk of lung cancer could be found in after PD group, but could not be found in before PD group. On the other hand, different roles which smoking plays in PD and lung cancer may be another reason. Nicotine was reported to be as a possible neuro-protective agent in patients with PD [24]. Recent studies reported that smoking was involved in the decreased risk of PD and the increased risk of lung cancer [9, 10]. Thus, it may also partly explain the significantly inverse association of PD and the risk of lung cancer.

Among the included studies in this study, only the studies of Minami [11] and Lin [7] were performed in Asian population. The result of study by Lin et al. showed that PD was significantly associated with an increased risk of lung cancer. This finding nearly contrary to of those in most cohort studies performed in Western populations. In other words, the results based on Western population may not be applied to Asian population. Different genetic backgrounds and habit and/or environmental factors combined may lead to the different results [7]. For instance, it was well-known that EGFR-activating mutations made patients more likely to suffer from lung cancer. The percentage of EGFR-activating mutations in lung cancer is about 50% in East Asian patients, but only 10% in White patients [25, 26]. Until now, there was no direct evidence to prove the relationship between EGFR mutations of lung cancer and PD. However, EGFR was reported to related with PD-related genes such as PARK2 [27], whose mutation was proved to be associated with lung cancer [28]. Thus, these results may provide a possible linkage between EGFR mutations and lung cancer risk in patients with PD. On the other hand, environmental factor such as pesticide exposure was reported to be associated with etiologies of PD [29] and the risk of lung cancer [30]. The different relationship between pesticide exposures and PD in different geographic areas may also partly explain this discrepancy [31, 32]. This dispute needs to be solved by future studies.

There are several strengths in this study. This study is the first meta-analysis which included 15 studies comprising 348,780 patients with PD in this field. Moreover, the results of this study are still robust as performed by further subgroup analysis, sensitivity analysis and cumulative meta-analysis. Certainly, some limitations exist in this meta-analysis. First, significant heterogeneity can be found in this study. Different population groups may be the possible explanation for the heterogeneity among the included studies. Second, the included studies in this study were retrospective cohort or case-control studies. Third, we cannot perform other subgroup analyses owing to limited data in the relevant studies.

## CONCLUSIONS

In summary, the result of this study indicated that patients with PD were significantly associated with a reduced risk of lung cancer in Western population. Future studies are warranted to confirm the association between them in Asian population.

## MATERIALS AND METHODS

### Literature search strategy

A comprehensive article search for relevant studies published was performed using the following online databases: PubMed, Web of Science and Embase. The main terms of “Parkinson's disease/Parkinson disease” and “Lung cancer” were used for relevant studies published up to August 31, 2016. Moreover, references of relevant reviews or included articles were also carefully reviewed to identify potential studies.

### Study selection and data extraction

The eligible studies were included in our meta-analysis on the basis of the following criteria: (1) cohort and/or case-control study evaluating the relationship between PD and risk of lung cancer; (2) an estimate of association [e.g. incidence rate ratio, odds ratio, risk ratio (RR), hazard ratio or standardized incidence ratio] with measures of variation (i.e. confidence intervals, CI) was provided; (3) published study in English. When duplicated studies were based on the same population, only the most informative study was included [7, 18]. Case reports and abstracts and from meetings were excluded.

Data of each study was extracted independently by two authors (Xin Xie and Xiaoguang Luo) according to the Preferred Reporting Items for Systematic Reviews and Meta-Analyses (PRISMA) guidelines (Table S1) [33], and any disagreements were resolved by discussion. The following data was extracted from each included study: the first author, population country, study design (cohort or case-control), publication year, patient information (i.e. sample size, source, age and sex), follow-up period and outcome of interest. The Newcastle-Ottawa scale (NOS) was used to assess the quality of included studies [34]. Study with NOS scores > 6 (median score) was assigned as high quality study (Table S2).

### Statistical analysis

The pooled RR and their 95% confidence intervals (CI) were calculated using the method of inverse variance with the random-effects model. Statistical heterogeneity was evaluated using Cochran's *Q* test and *I*^2^ statistics [35]. Begg's and Egger's tests were used to evaluate the effect of publication bias [36, 37]. Cumulative meta-analysis was used to assess the evolution of the combined RR with relation to the year of publication [38].

STATA software (version 12.0; Stata Corporation, College Station, TX, USA) was used to perform the data analyses in this study. It was considered statistically significant when *P* value was less than 0.05.

## SUPPLEMENTARY MATERIALS METHODS TABLE AND FIGURES




