# Supplementary material for: Risk of lung cancer in Parkinson's disease
Source: Oncotarget. 2016 Oct 27;7(47):77319–25. doi: 10.18632/oncotarget.12964 (PMC5363588; doi:10.18632/oncotarget.12964)
Supplement: Supplementary file 1 [file oncotarget-07-77319-s001.pdf]

Risk of lung cancer in Parkinson's disease

Supplementary Materials

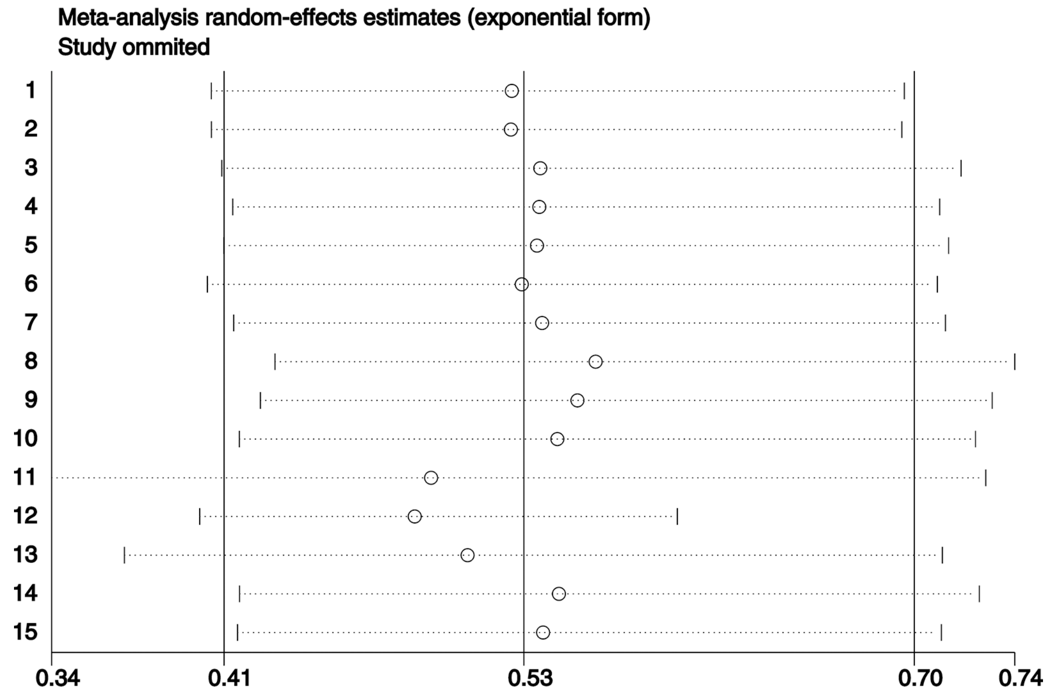

Supplementary Figure S1: Sensitivity analysis via elimination of each study in turn.

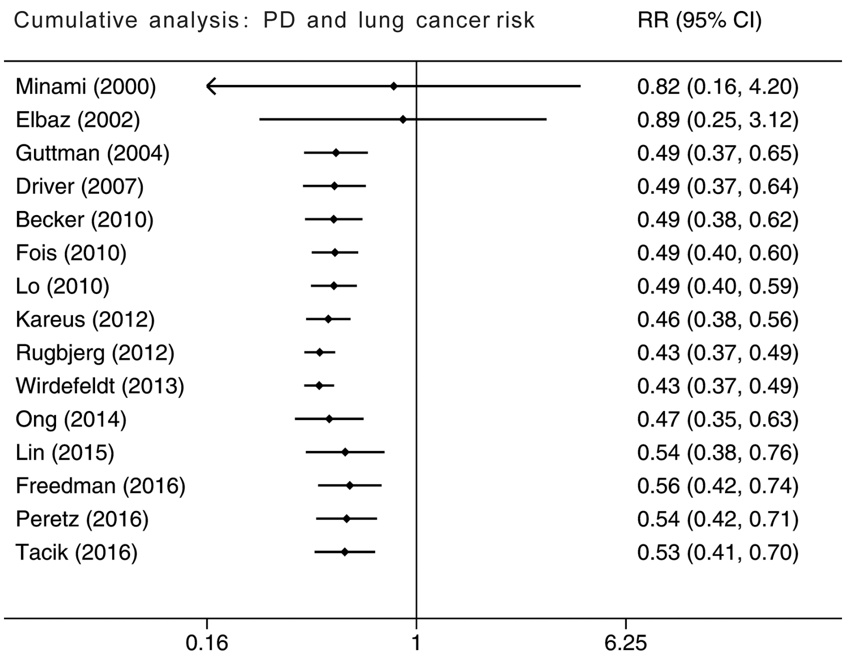

Supplementary Figure S2: Cumulative meta-analysis.

List of excluded studies

Studies were excluded because of not relevant data

1. Ramaiah S, Ganesan R, Mangham DC, McNally O, Klys HS, Hirschowitz L. Malignant variant of sclerosing perivascular epithelioid cell tumor arising in the adnexa. Int J Gynecol Pathol. 2009; 28:589–593.
2. Fujino I. [EFFECTS OF HYPERCAPNIA UPON THE PULMONARY ARTERY PRESSURE]. Jpn Circ J. 1963; 27:835–851.
3. Boiteau GM, Bourassa MG. [CLINICAL EVALUATION OF INCOMPLETE RIGHT BRANCH BLOCK]. Union Med Can. 1964; 93:842–845.
4. Forno LS. Concentric hyalin intraneuronal inclusions of Lewy type in the brains of elderly persons (50 incidental cases): relationship to parkinsonism. J Am Geriatr Soc. 1969; 17:557–575.
5. Westlund K. Distribution and mortality time trend of multiple sclerosis and some other diseases in Norway. Acta Neurol Scand. 1970; 46:455–483.
6. Kessler, II. Epidemiologic studies of Parkinson's disease. II. A hospital-based survey. Am J Epidemiol. 1972; 95:308–318.
7. Chou TC. Pseudo-infarction (noninfarction Q waves). Cardiovasc Clin. 1973; 5:199–218.
8. Pritchard PB, 3rd and Netsky MG. Prevalence of neoplasms and causes of death in paralysis agitans. A necropsy study. Neurology. 1973; 23:215–222.
9. Hatake S, Masuyama Y, Oda T, Ishii T, Kimoto B. [Tremor, agnosia, a stooping posture and pyrexia (thoracic radiography and EEG): (bronchial cancer--squamous cell carcinoma--and parkinsonism)]. Cardiovasc Clin. 1977; 35:3064–3065, 3428–3069.
10. Carolo F, Canton C, Orcalli F, Fiaschi A, Tomelleri G, Puchetti V. [Lipoid pulmonary granuloma in Parkinson's disease. Presentation of clinical case]. Chir Ital. 1979; 31:1314–1320.
11. Wildbolz A. [Diagnostic errors due to unilateral psychiatric or unilateral somatic appreciation (author's transl)]. Schweiz Rundsch Med Prax. 1979; 68:1482–1487.
12. Rogot E, Murray JL. Smoking and causes of death among U.S. veterans: 16 years of observation. Public Health Rep. 1980; 95:213–222.
13. Weishaar RE, Cain MH, Bristol JA. A new generation of phosphodiesterase inhibitors: multiple molecular forms of phosphodiesterase and the potential for drug selectivity. J Med Chem. 1985; 28:537–545.
14. Dutcher JP, Creekmore S, Weiss GR, Margolin K, Markowitz AB, Roper M, Parkinson D, Ciobanu N, Fisher RI, Boldt DH and et al. A phase II study of interleukin-2 and lymphokine-activated killer cells in patients with metastatic malignant melanoma. J Clin Oncol. 1989; 7:477–485.
15. Isozaki E, Miyamoto K, Tanabe H, Oda M. [Morphological changes in human diaphragm--ragged red fiber, core/targetoid fiber, cytoplasmic body, and ring fiber]. Rinsho shinkeigaku. 1989; 29:726–733.
16. Wrensch MR, Barger GR. Familial factors associated with malignant gliomas. Genetic epidemiology. 1990; 7:291–301.
17. Riggs JE. The decline of mortality due to stroke: a competitive and deterministic perspective. Neurology. 1991; 41:1335–1338.
18. Yang SC, Grimm EA, Parkinson DR, Carinhas J, Fry KD, Mendiguren-Rodriguez A, Licciardello J, Owen-Schaub LB, Hong WK, Roth JA. Clinical and immunomodulatory effects of combination immunotherapy with low-dose interleukin 2 and tumor necrosis factor alpha in patients with advanced non-small cell lung cancer: a phase I trial. Cancer Res. 1991; 51:3669–3676.
19. Mera SL. Senescence and pathology in ageing. Med Lab Sci. 1992; 49:271–282.
20. Lilienfeld DE, Perl DP. Projected neurodegenerative disease mortality in the United States, 1990–2040. Neuroepidemiology. 1993; 12:219–228.
21. Daly AK, Cholerton S, Armstrong M, Idle JR. Genotyping for polymorphisms in xenobiotic metabolism as a predictor of disease susceptibility. Environ Health Perspect. 1994; 102 Suppl 9:55–61.
22. Doll R, Peto R, Wheatley K, Gray R, Sutherland I. Mortality in relation to smoking: 40 years' observations on male British doctors. BMJ. 1994; 309:901–911.
23. Gorell JM, Johnson CC, Rybicki BA. Parkinson's disease and its comorbid disorders: an analysis of Michigan mortality data, 1970 to 1990. Neurology. 1994; 44:1865–1868.
24. Lilienfeld DE, Perl DP. Projected neurodegenerative disease mortality among minorities in the United States, 1990–2040. Neuroepidemiology. 1994; 13:179–186.
25. Maffioli L, Mascheroni L, Mongioj V, Gasparini M, Baldini MT, Seregni E, Castellani MR, Cascinelli N, Buraggi GL. Scintigraphic detection of melanoma metastases with a radiolabeled benzamide ([iodine-123]-(S)-IBZM). J Nucl Med. 1994; 35:1741–1747.
26. Cullen MH, Stenning SP, Parkinson MC, Fossa SD, Kaye SB, Horwich AH, Harland SJ, Williams MV, Jakes R. Short-course adjuvant chemotherapy in high-risk stage I nonseminomatous germ cell tumors of the testis: a Medical Research Council report. J Clin Oncol. 1996; 14:1106–1113.
27. Tsuneoka Y, Fukushima K, Matsuo Y, Ichikawa Y, Watanabe Y. Genotype analysis of the CYP2C19 gene in the Japanese population. Life Sci. 1996; 59:1711–1715.
28. Kakkar R, Raju RV, Rajput AH, Sharma RK. Amantadine: an antiparkinsonian agent inhibits bovine brain 60 kDa calmodulin-dependent cyclic nucleotide phosphodiesterase isozyme. Brain Res. 1997; 749:290–294.
29. Miyashita N, Kondo T, Wakiya M, Mori H, Shirai T, Takubo H, Mizuno Y. [A 61-year-old man with progressive gait disturbance, freezing, and vertical gaze paresis who developed esophagus cancer]. No to shinkei. 1998; 50:1041–1052.
30. Ried LD, Johnson RE, Gettman DA. Benzodiazepine exposure and functional status in older people. J Am Geriatr Soc. 1998; 46:71–76.

31. Rostami-Hodjegan A, Lennard MS, Woods HF, Tucker GT. Meta-analysis of studies of the CYP2D6 polymorphism in relation to lung cancer and Parkinson's disease. Pharmacogenetics. 1998; 8:227–238.
32. Hochster H, Strawderman MH, Harris JE, Atkins MB, Oken M, Skeel RT, Jubelirer SJ, Parkinson D. Conventional dose melphalan is inactive in metastatic melanoma: results of an Eastern Cooperative Oncology Group Study (E1687). Anti-cancer drugs. 1999; 10:245–248.
33. Konagaya Y, Ohase M. [Hemiparkinsonism associated with metastatic brain tumour]. No to shinkei. 1999; 51:820–821.
34. Bagchi D, Bagchi M, Stohs SJ, Das DK, Ray SD, Kuszynski CA, Joshi SS, Pruess HG. Free radicals and grape seed proanthocyanidin extract: importance in human health and disease prevention. Toxicology. 2000; 148:187–197.
35. Higes-Pascual F, Beroiz-Groh P, Bravo-Guillen AI, Palomo-Martinez V, Cuevas-Santos J. [Leptomeningeal carcinomatosis as presenting symptom of a gallbladder carcinoma]. Rev Neurol. 2000; 30:841–844.
36. Phelps ME. Positron emission tomography provides molecular imaging of biological processes. Proc Natl Acad Sci U S A. 2000; 97:9226–9233.
37. Phelps ME. PET: the merging of biology and imaging into molecular imaging. J Nucl Med. 2000; 41:661–681.
38. Furet Y, Bechtel Y, Le Guellec C, Bechtel PR, Autret-Leca E, Paintaud G. [Clinical relevance of N-acetyltransferase type 2 (NAT2) genetic polymorphism]. Therapie. 2002; 57:427–431.
39. Cesari R, Martin ES, Calin GA, Pentimalli F, Bichi R, McAdams H, Trapasso F, Drusco A, Shimizu M, Masciullo V, D'Andrilli G, Scambia G, Picchio MC, et al. Parkin, a gene implicated in autosomal recessive juvenile parkinsonism, is a candidate tumor suppressor gene on chromosome 6q25-q27. Proc Natl Acad Sci U S A. 2003; 100:5956–5961.
40. Thapar GD. Approach to the problems of the aged. J Indian Med Assoc. 2004; 102:93–96.
41. Nakajima M, Yokoi T. Interindividual variability in nicotine metabolism: C-oxidation and glucuronidation. Drug Metab Pharmacokinet. 2005; 20:227–235.
42. Chan JM, Feraco A, Shuman M, Hernandez-Diaz S. The epidemiology of prostate cancer—with a focus on nonsteroidal anti-inflammatory drugs. Hematol Oncol Clin North Am. 2006; 20:797–809.
43. Denning GM, Stoll LL. Peroxisome proliferator-activated receptors: potential therapeutic targets in lung disease? Pediatr Pulmonol. 2006; 41:23–34.
44. Hofmann J, Guardado J, Keifer M, Wesseling C. Mortality among a cohort of banana plantation workers in Costa Rica. Int J Occup Environ Health. 2006; 12:321–328.
45. Kannan S, Wu M. Respiratory stem cells and progenitors: overview, derivation, differentiation, carcinogenesis, regeneration and therapeutic application. Curr Stem Cell Res Ther. 2006; 1:37–46.
46. Daly JW. Caffeine analogs: biomedical impact. Cell Mol Life Sci. 2007; 64:2153–2169.
47. Prelipcean CC, Mihai C, Gogalniceanu P, Mitrica D, Drug VL, Stanciu C. Extragastric manifestations of Helicobacter pylori infection. Rev Med Chir Soc Med Nat Iasi. 2007; 111:575–583.
48. VanBrocklin HF, Hanrahan SM, Enas JD, Nandanan E, O'Neil JP. Mitochondrial avid radioprobes. Preparation and evaluation of 7'(Z)-[125I]iodorotenone and 7'(Z)-[125I] iodorotenol. Nucl Med Biol. 2007; 34:109–116.
49. Jorgensen E, Stinson A, Shan L, Yang J, Gietl D, Albino AP. Cigarette smoke induces endoplasmic reticulum stress and the unfolded protein response in normal and malignant human lung cells. BMC cancer. 2008; 8:229.
50. Kim WY, Chang DJ, Hennessy B, Kang HJ, Yoo J, Han SH, Kim YS, Park HJ, Seo SY, Mills G, Kim KW, Hong WK, Suh YG, et al. A novel derivative of the natural agent deguelin for cancer chemoprevention and therapy. Cancer Prev Res (Phila). 2008; 1:577–587.
51. Mimeault M, Batra SK. Recent progress on tissue-resident adult stem cell biology and their therapeutic implications. Stem cell reviews. 2008; 4:27–49.
52. Morozova N, O'Reilly EJ, Ascherio A. Variations in gender ratios support the connection between smoking and Parkinson's disease. Mov Disord. 2008; 23:1414–1419.
53. Neumann H, Hazen JL, Weinstein J, Mehl RA, Chin JW. Genetically encoding protein oxidative damage. J Am Chem Soc. 2008; 130:4028–4033.
54. Gao X, Simon KC, Han J, Schwarzschild MA, Ascherio A. Family history of melanoma and Parkinson disease risk. Neurology. 2009; 73:1286–1291.
55. Kim JS, Yim SV, Koh IS, Choi JS, Yoo JY, Lee KS, Lim Y, Lee KH. Single-nucleotide polymorphisms (SNPs) and haplotype analysis in vascular endothelial growth factor (VEGF) gene in the patients with Parkinson disease and lung cancer. Arch Gerontol Geriatr. 2009; 48:287–290.
56. Roberts RA, Laskin DL, Smith CV, Robertson FM, Allen EM, Doorn JA, Slikker W. Nitrative and oxidative stress in toxicology and disease. Toxicol Sci. 2009; 112:4–16.
57. Hippisley-Cox J, Coupland C. Unintended effects of statins in men and women in England and Wales: population based cohort study using the QResearch database. BMJ. 2010; 340:c2197.
58. Shadvar A, Erfanian A. Mutual information-based Fisher discriminant analysis for feature extraction and recognition with applications to medical diagnosis. Annual International Conference of the IEEE Engineering in Medicine and Biology Society IEEE Engineering in Medicine and Biology Society Annual Conference. 2010; 2010:5811–5814.
59. Tikhmyanova N, Little JL, Golemis EA. CAS proteins in normal and pathological cell growth control. Cell Mol Life Sci: CMLS. 2010; 67:1025–1048.
60. Tisato F, Marzano C, Porchia M, Pellei M, Santini C. Copper in diseases and treatments, and copper-based anticancer strategies. Med Res Rev. 2010; 30:708–749.

61. Berthier A, Navarro S, Jimenez-Sainz J, Rogla I, Ripoll F, Cervera J, Pulido R. PINK1 displays tissue-specific subcellular location and regulates apoptosis and cell growth in breast cancer cells. Hum Pathol. 2011; 42:75–87.

62. Akushevich I, Kravchenko J, Ukraintseva S, Arbeev K, Yashin AI. Age patterns of incidence of geriatric disease in the U.S. elderly population: J Am Geriatr Soc. 2012; 60:323–327.

63. Palma E, Conti L, Roseti C, Limatola C. Novel approaches to study the involvement of alpha7-nAChR in human diseases. Curr Drug Targets. 2012; 13:579–586.

64. Payne C, Wiffen PJ, Martin S. Interventions for fatigue and weight loss in adults with advanced progressive illness. Cochrane Database Syst Rev. 2012; 1:Cd008427.

65. Akushevich I, Kravchenko J, Ukraintseva S, Arbeev K, Kulminski A, Yashin AI. Morbidity risks among older adults with pre-existing age-related diseases. Exp Gerontol. 2013; 48:1395–1401.

66. Akushevich I, Kravchenko J, Ukraintseva S, Arbeev K, Yashin AI. Time trends of incidence of age-associated diseases in the US elderly population: Medicare-based analysis. Age Ageing. 2013; 42:494–500.

67. Diestre Ortin G, Gonzalez Sequero V, Collell Domenech N, Perez Lopez F, Hernando Robles P. [Advance care planning and severe chronic diseases]. Rev Esp Geriatr Gerontol. 2013; 48:228–231.

68. Esseltine JL, Willard MD, Wulur IH, Lajiness ME, Barber TD, Ferguson SS. Somatic mutations in GRM1 in cancer alter metabotropic glutamate receptor 1 intracellular localization and signaling. Mol Pharmacol. 2013; 83:770–780.

69. Mulligan VK, Chakrabartty A. Protein misfolding in the late-onset neurodegenerative diseases: common themes and the unique case of amyotrophic lateral sclerosis. Proteins. 2013; 81:1285–1303.

70. Betsou F. Clinical biospecimens: reference materials, certified for nominal properties? Biopreserv Biobank. 2014; 12:113–120.

71. Chandran G, Muralidhara. Insights on the neuromodulatory propensity of Selaginella (Sanjeevani) and its potential pharmacological applications. CNS Neurol Disord Drug Targets. 2014; 13:82–95.

72. Domanskyi A, Alter H, Vogt MA, Gass P, Vinnikov IA. Transcription factors Foxa1 and Foxa2 are required for adult dopamine neurons maintenance. Front Cell Neurosci. 2014; 8:275.

73. Forester SC, Lambert JD. Synergistic inhibition of lung cancer cell lines by (−)-epigallocatechin-3-gallate in combination with clinically used nitrocatechol inhibitors of catechol-O-methyltransferase. Carcinogenesis. 2014; 35:365–372.

74. Ibanez K, Boullosa C, Tabares-Seisdedos R, Baudot A, Valencia A. Molecular evidence for the inverse comorbidity between central nervous system disorders and cancers detected by transcriptomic meta-analyses. PLoS Genet. 2014; 10:e1004173.

75. Kim B, Sohn EJ, Jung JH, Shin EA, You OH, Im J, Kim SH. Inhibition of ZNF746 suppresses invasion and epithelial to mesenchymal transition in H460 non-small cell lung cancer cells. Oncol Rep. 2014; 31:73–78.

76. Kim HJ, Kim HJ, Jeong JE, Baek JY, Jeong J, Kim S, Kim YM, Kim Y, Nam JH, Huh SH, Seo J, Jin BK, Lee KJ. N-terminal truncated UCH-L1 prevents Parkinson's disease associated damage. PloS one. 2014; 9:e99654.

77. Lu W, Karuppagounder SS, Springer DA, Allen MD, Zheng L, Chao B, Zhang Y, Dawson VL, Dawson TM, Lenardo M. Genetic deficiency of the mitochondrial protein PGAM5 causes a Parkinson's-like movement disorder. Nat Commun. 2014; 5:4930.

78. Mittal D, Young A, Stannard K, Yong M, Teng MW, Allard B, Stagg J, Smyth MJ. Antimetastatic effects of blocking PD-1 and the adenosine A2A receptor. Cancer Res. 2014; 74:3652–3658.

79. Moreno B, Lopez I, Fernandez-Diez B, Gottlieb M, Matute C, Sanchez-Gomez MV, Domercq M, Giralt A, Alberch J, Collon KW, Zhang H, Parent JM, Teixido M, et al. Differential neuroprotective effects of 5′-deoxy-5′-methylthioadenosine. PloS one. 2014; 9:e90671.

80. Agarwal S, Mishra P, Shivange G, Kodipelli N, Moros M, de la Fuente JM, Anindya R. Citrate-capped gold nanoparticles for the label-free detection of ubiquitin C-terminal hydrolase-1. The Analyst. 2015; 140:1166–1173.

81. Do R, Stitziel NO, Won HH, Jorgensen AB, Duga S, Angelica Merlini P, Kiezun A, Farrall M, Goel A, Zuk O, Guella I, Asselta R, Lange LA, et al. Exome sequencing identifies rare LDLR and APOA5 alleles conferring risk for myocardial infarction. Nature. 2015; 518:102–106.

82. Gamus D. [Advances in research of complementary and integrative medicine: a review of recent publications in some of the leading medical journals]. Harefuah. 2015; 154:9–15, 70.

83. Garcia-Carrasco M, Mendoza-Pinto C, Macias Diaz S, Vera-Recabarren M, Vazquez de Lara L, Mendez Martinez S, Soto-Santillan P, Gonzalez-Ramirez R, Ruiz-Arguelles A. P-glycoprotein in autoimmune rheumatic diseases. Autoimmun Rev. 2015; 14:594–600.

84. Koyama S, Omura T, Yonezawa A, Imai S, Nakagawa S, Nakagawa T, Yano I, Matsubara K. Gefitinib and Erlotinib Lead to Phosphorylation of Eukaryotic Initiation Factor 2 Alpha Independent of Epidermal Growth Factor Receptor in A549 Cells. PloS one. 2015; 10:e0136176.

85. Lawrence KM, Jackson TR, Jamieson D, Stevens A, Owens G, Sayan BS, Locke IC, Townsend PA. Urocortin—from Parkinson's disease to the skeleton. Int J Biochem Cell Biol. 2015; 60:130–138.

86. Leone RD, Lo YC, Powell JD. A2aR antagonists: Next generation checkpoint blockade for cancer immunotherapy. Comput Struct Biotechnol J. 2015; 13:265–272.

87. Liu QX, Zheng H, Deng XF, Zhou D, Dai JG. Status of the Parkinson's disease gene family expression in non-small-cell lung cancer. World J Surg Oncol. 2015; 13:238.

88. Pedersen BK, Saltin B. Exercise as medicine - evidence for prescribing exercise as therapy in 26 different chronic diseases. Scand J Med Sci Sports. 2015; 25 Suppl 3:1–72.

89. Sheinerman KS, Umansky S. Universal screening test based on analysis of circulating organ-enriched microRNAs: a novel approach to diagnostic screening. Expert Rev Mol Diagn. 2015; 15:329–338.

90. Threadgill MD. 5-Aminoisoquinolin-1-one (5-AIQ), a Water-Soluble Inhibitor of the Poly(ADP-Ribose)Polymerases (PARPs). Curr Med Chem. 2015; 22:3807–3829.

91. Vavougios GD, Solenov EI, Hatzoglou C, Baturina GS, Katkova LE, Molyvdas PA, Gourgoulianis KI, Zarogiannis SG. Computational genomic analysis of PARK7 interactome reveals high BBS1 gene expression as a prognostic factor favoring survival in malignant pleural mesothelioma. Am J Physiol Lung Cell Mol Physiol. 2015; 309:L677–686.

92. Xiong D, Wang Y, Kupert E, Simpson C, Pinney SM, Gaba CR, Mandal D, Schwartz AG, Yang P, de Andrade M, Pikielny C, Byun J, Li Y, et al. A recurrent mutation in PARK2 is associated with familial lung cancer. Am J Hum Genet. 2015; 96:301–308.

93. Zhang D, Wang L, Yan L, Miao X, Gong C, Xiao M, Ni R, Tang Q. Vacuolar protein sorting 4B regulates apoptosis of intestinal epithelial cells via p38 MAPK in Crohn's disease. Exp Mol Pathol. 2015; 98:55–64.

94. Ajdacic-Gross V, Rodgers S, Aleksandrowicz A, Mutsch M, Steinemann N, von Wyl V, von Kanel R, Bopp M. Cancer co-occurrence patterns in Parkinson's disease and multiple sclerosis-Do they mirror immune system imbalances? Cancer Epidemiol. 2016; 44:167–173.

95. Gavett SH, Parkinson CU, Willson GA, Wood CE, Jarabek AM, Roberts KC, Kodavanti UP, Dodd DE. Persistent effects of Libby amphibole and amosite asbestos following subchronic inhalation in rats. Part Fibre Toxicol. 2016; 13:17.

96. Klionsky DJ, Abdelmohsen K, Abe A, Abedin MJ, Abeliovich H, Acevedo Arozena A, Adachi H, Adams CM, Adams PD, Adeli K, Adhihetty PJ, Adler SG, Agam G, et al. Guidelines for the use and interpretation of assays for monitoring autophagy (3rd edition). Autophagy. 2016; 12:1–222.

97. Oliveira C, Ribeiro AJ, Veiga F, Silveira I. Recent Advances in Nucleic Acid-Based Delivery: From Bench to Clinical Trials in Genetic Diseases. J Biomed Nanotechnol. 2016; 12:841–862.

Studies did not meet inclusion criteria

1. Bajaj A, Driver JA, Schernhammer ES. Parkinson's disease and cancer risk: a systematic review and meta-analysis. Cancer Causes Control: 2010; 21:697–707.

2. Catala-Lopez F, Suarez-Pinilla M, Suarez-Pinilla P, Valderas JM, Gomez-Beneyto M, Martinez S, Balanza-Martinez V, Climent J, Valencia A, McGrath J, Crespo-Facorro B, Sanchez-Moreno J, Vieta E, et al. Inverse and direct cancer comorbidity in people with central nervous system disorders: a meta-analysis of cancer incidence in 577,013 participants of 50 observational studies. Psychother Psychosom. 2014; 83:89–105.

3. Jansson B, Jankovic J. Low cancer rates among patients with Parkinson's disease. Ann Neurol. 1985; 17:505–509.

4. Beyer MK, Herlofson K, Arsland D, Larsen JP. Causes of death in a community-based study of Parkinson's disease. Acta Neurol Scand. 2001; 103:7–11.

5. D'Amelio M, Ragonese P, Sconzo G, Aridon P, Savettieri G. Parkinson's disease and cancer: insights for pathogenesis from epidemiology. Ann N Y Acad Sci. 2009; 1155:324–334.

6. Garber K. Parkinson's disease and cancer: the unexplored connection. J Natl Cancer Inst. 2010; 102:371–374.

7. Devine MJ, Plun-Favreau H, Wood NW. Parkinson's disease and cancer: two wars, one front. Nat Rev Cancer. 2011; 11:812–823.

8. Korkmaz T, Seber S, Basaran G, Yumuk PF, Dane F, Kocar M, Telli F, Turhal NS. 5-Flourouracil-Induced Encephalopathy in Parkinson's Disease. Uhod-Uluslar Hematol. 2012; 22:51–53.

9. Lethbridge L, Johnston GM, Turnbull G. Co-morbidities of persons dying of Parkinson's disease. Prog Palliat Care. 2013; 21:140–145.

10. Chen H, Ding D, Wang J, Zhao Q, Meng H, Li H, Gao YT, Shu XO, Tanner CM, Hong Z, Yang G. Parkinson's disease research in a prospective cohort in China. Parkinsonism Relat Disord. 2015; 21:1200–1204.

11. Moens K, Houttekier D, Van den Block L, Harding R, Morin L, Marchetti S, Csikos A, Loucka M, Naylor WA, Wilson DM, Teno J, Cardenas-Turanzas M, Rhee Y, et al. Place of death of people living with Parkinson's disease: a population-level study in 11 countries. BMC Palliat Care. 2015; 14:28.

Studies based on the same population

1. Moller H, Mellemkjaer L, McLaughlin JK, Olsen JH. Occurrence of different cancers in patients with Parkinson's disease. BMJ. 1995; 310:1500–1501.

2. Elbaz A, Peterson BJ, Bower JH, Yang P, Maraganore DM, McDonnell SK, Ahlskog JE, Rocca WA. Risk of cancer after the diagnosis of Parkinson's disease: a historical cohort study. Mov Disord. 2005; 20:719–725.

3. Olsen JH, Friis S, Frederiksen K, McLaughlin JK, Mellemkjaer L, Moller H. Atypical cancer pattern in patients with Parkinson's disease. Br J Cancer. 2005; 92:201–205.

4. Olsen JH, Friis S, Frederiksen K. Malignant melanoma and other types of cancer preceding Parkinson disease. Epidemiology. 2006; 17:582–587.

5. Sun LM, Liang JA, Chang SN, Sung FC, Muo CH, Kao CH. Analysis of Parkinson's disease and subsequent cancer risk in Taiwan: a nationwide population-based cohort study. Neuroepidemiology. 2011; 37:114–119.
6. Lai SW, Liao KF, Lin CH, Tsai PY, Sung FC. Parkinson's disease and lung cancer: a population-based case-control study in Taiwan. Geriatr Gerontol Int. 2013; 13:238–240.
7. Peretz C, Chillag-Talmor O, Linn S, Gurevich T, El-Ad B, Silverman B, Friedman N, Giladi N. Parkinson's disease patients first treated at age 75 years or older: a comparative study. Parkinsonism Relat Disord. 2014; 20:69–74.

Supplementary Table S1: PRISMA Checklist. See Supplementary_Table_S1.

Supplementary Table S2: The NOS quality of included studies

Study	Selection				Comparability		Outcome/Exposure			Total	Quality
	REC/ ICDA	SNEC/ RC	AE/ SOC	DO/ DC	SC	AF	AO	FU/ SMACC	AFU/ NRR		
Cohort											
Minami 2000	1	0	1	1	0	0	1	1	0	5	Low
Guttman 2004	1	0	1	1	1	0	1	1	0	6	Low
Driver 2007	1	1	1	1	1	0	0	1	0	6	Low
Becker 2010	1	1	1	1	1	1	0	1	0	7	High
Foris 2010	1	0	1	1	1	1	1	1	0	7	High
Lo 2010	1	1	1	1	1	1	1	0	0	7	High
Kareus 2012	1	0	1	1	1	0	0	1	0	5	Low
Rugbjerg 2015	1	0	1	1	1	0	1	1	1	7	High
Wirdefeldt 2015	1	1	1	1	1	0	1	1	0	7	High
Ong 2014	1	1	1	1	1	0	1	1	1	8	High
Lin 2015	1	1	1	1	1	0	1	1	0	7	High
Petrez 2016	1	0	1	0	1	0	1	1	1	6	Low
Case-control											
Elbaz 2002	1	1	1	1	1	0	1	1	0	7	High
Freedman 2016	1	1	0	0	1	0	1	1	0	5	Low
Tacik 2016	1	1	1	0	0	0	1	1	0	5	Low

Abbreviations, AE: ascertainment of exposure; AF: study controls for any additional factors; AFU: adequacy of follow-up of cohorts; AO: assessment of outcome; DO: demonstration that outcome of interest was not present at start of study; FU: follow-up long enough (36 months) for outcomes to occur; ICDA: Is the case definition adequate; RC: Representativeness of the cases; REC: representativeness of the exposed cohort; SC: study controls for age, sex; "1" means that the study is satisfied the item and "0" means the opposite situation; SNEC: selection of the non-exposed cohort; SOC: Selection of controls; DC: Definition of controls; SMACC: Same method of ascertainment for cases and control; NRR: Non-Response rate.
